# How to be a dioecious fig: Chemical mimicry between sexes matters only when both sexes flower synchronously

**DOI:** 10.1038/srep21236

**Published:** 2016-02-18

**Authors:** M. Hossaert-McKey, M. Proffit, C. C. L. Soler, C. Chen, J.-M. Bessière, B. Schatz, R. M. Borges

**Affiliations:** 1 Centre d’Ecologie Fonctionnelle et Evolutive (CEFE), UMR 5175, CNRS—Université de Montpellier—Université Paul Valéry Montpellier—EPHE, 1919 route de Mende, 34293 Montpellier, France; 2 Xishuangbanna Tropical Botanical Garden (XTBG); National Chinese Academy of Sciences; Menglun, Yunnan China;; 3 Laboratoire de Chimie Appliquée, Ecole Nationale Supérieure de Chimie de Montpellier, 8 rue de l’Ecole Normale, 34296 Montpellier Cedex 5, France; 4 Centre for Ecological Sciences, Indian Institute of Science, Bangalore 560 012, India

## Abstract

In nursery pollination mutualisms, which are usually obligate interactions, olfactory attraction of pollinators by floral volatile organic compounds (VOCs) is the main step in guaranteeing partner encounter. However, mechanisms ensuring the evolutionary stability of dioecious fig–pollinator mutualisms, in which female fig trees engage in pollination by deceit resulting in zero reproductive success of pollinators that visit them, are poorly understood. In dioecious figs, individuals of each sex should be selected to produce odours that their pollinating wasps cannot distinguish, especially since pollinators have usually only one choice of a nursery during their lifetime. To test the hypothesis of intersexual chemical mimicry, VOCs emitted by pollen-receptive figs of seven dioecious species were compared using headspace collection and gas chromatography-mass spectrometry analysis. First, fig-flower scents varied significantly among species, allowing host-species recognition. Second, in species in which male and female figs are synchronous, intersexual VOC variation was not significant. However, in species where figs of both sexes flower asynchronously, intersexual variation of VOCs was detectable. Finally, with one exception, there was no sexual dimorphism in scent quantity. We show that there are two ways to use scent to be a dioecious fig based on differences in flowering synchrony between the sexes.

Among the sexual strategies to avoid inbreeding in flowering plants, sexual dimorphism promotes the evolution of dioecy^1^. The transition from monoecy to dioecy in plants is an important event in the evolution of angiosperms^2^, with dioecious species occurring in half of all plant families but representing only 6–7% of all species^3^. In clades with a large number of dioecious species, Vamosi and Vamosi^4^ found a higher extinction rate as well as a lower speciation rate than in their non-dioecious sister clades, a difference that may be explained by the strong sexual dimorphism displayed by dioecious species. Indeed, one of the consequences of such sexual specialization is that reward levels to pollinators often differ between male and female flowers as a result of sexual selection^5^. Such specialization leads, in some cases, to cheating by one deceptive sex, which can be either male or female^6^. In such deceptive pollination systems, signals are key factors in maintaining the interaction^7^. For the deceptive sex it is crucial to maintain signals that mimic the signals of the rewarding sex in order to attract pollinators and ensure mating success, thereby maximizing the fitness of both sexes. Among these signals, while floral scents (i.e. bouquets of volatile organic compounds (VOCs) emitted by flowers) are often crucial signals for pollinator attraction^8^,^9^, they may be among the most variable traits of the plant phenotype^10^. However, some cases of strong stabilizing selection on floral scent exist^11^,^12^. Despite the potential for variable evolutionary trajectories, floral scents in dioecious species have been much less studied than other floral traits in the context of sexual dimorphism^5^.

In nursery pollination mutualisms, which are highly species-specific interactions where reproduction of the partners is completely inter-dependent, chemical signals are crucial in partner encounter^13^. To ensure effective partner encounter, selection should act both on the specific volatile signals emitted by the host plant and on the capacity of the pollinating insect to detect their message, ensuring effective communication. More than half of the nursery pollination systems described so far involve dioecious species^13^,^14^. They thus provide excellent systems to study the constraints imposed on plants and pollinators, as well as the strong necessity for inter-sexual resemblance in these signals when the sexes are separate and offer different rewards to their pollinators. In fact, there are many dioecious species where the chemical composition of scents of the rewardless sex – often the females – is similar to those of male plants, supporting the predictions of the intersexual mimicry hypothesis^5^,^15^. However, sexual dimorphism in scent composition also exists in several dioecious species^5^. In addition, dimorphism in floral scents may also be due to variation in the quantity of emitted VOCs with one sex, very often the males, having higher VOC emission rates^5^.

With more than 300 dioecious species, the genus *Ficus* (Moraceae) provides an excellent opportunity to test the intersexual mimicry hypothesis of floral scents in nursery pollination mutualisms, since all *Ficus* display strategies of pollinator attraction strongly based on chemical mediation. Nearly every fig species is usually obligatorily pollinated by a single species of fig wasp (Hymenoptera, Chalcidoidea: Agaonidae), which can only reproduce within the host fig^16^. The female pollinator wasps locate figs of the host by species-specific bouquets of VOC attractants produced by figs at receptivity, i.e. the developmental stage during which the stigmas are ready for pollination^17^,^18^,^19^. Dioecious figs represent a special case of pollination by deceit with fatal consequences for female wasps that enter female figs; such wasps will have zero reproductive success, for the following reasons. Dioecious fig species are anatomically gynodioecious, with true female trees and hermaphrodite but functionally male trees. Figs of male trees bear male and female flowers, but their short-styled female flowers serve only as brood sites for pollinators and never develop into seeds^16^,^20^. Female fig wasps enter male figs to lay eggs in the ovaries and then die. Some weeks later, the mature wasp offspring emerge and mate within the fig; the female wasps then exit their natal fig, loaded with pollen, and search for receptive figs in which to lay eggs. The male figs therefore produce pollen and pollinator wasps, but no seeds. The flowers of female figs, on the other hand, have long styles: female wasps can pollinate them but cannot lay eggs^21^. As a result, a pollinator wasp (which lives only for a few hours) entering a female fig dies without leaving any offspring; hence female figs produce only seeds. In other words, in the dioecious fig–pollinator wasp mutualism, the pollinating wasps pay a very high fitness price when entering a female fig. Thus, selection should favour wasps that choose to avoid female figs and only enter male figs (in which they reproduce). However, this “simple” behavioural shift in turn would lead to the end of fig seed production and to a breakdown of the dioecious system in figs^15^.

Different hypotheses have been erected to explain why this conflict has not caused the mutualism between fig wasps and fig trees to dissolve under dioecy^22^. One explanation for why wasps enter female figs is that, in some dioecious fig species, they have no option other than to enter female figs. In seasonally reproducing species such as *F. carica*^23^, male trees in a population are simultaneously receptive, but this is well before or after the synchronized receptivity of the female trees. Wasps that exit male figs during the time of synchronous female receptivity have no choice, and due to strong chemically mediated species-recognition mechanisms based on chemical signals^13^, they will be attracted to the deceptive female figs. The wasp population in these species is maintained by the emergence of a few wasps, at the end of the period of female flowering, which enter some of the few male figs that are receptive at that time and in which they can reproduce^20^,^24^.

However, in most dioecious fig species, male and female trees are frequently receptive at the same time (synchronous flowering), and the “no available choice” hypothesis does not apply. Several hypotheses have been proposed to explain the persistence of the mutualism in such dioecious fig species. Among them is “selection to rush”: sexual dimorphism could exist in some fig species, as in other dioecious species^5^, but due to the very short lifespan of the pollinating fig wasps^25^, they cannot afford the time to choose fig sex: thus, based on the strong host species recognition mechanism, they should enter the first fig encountered because any extra time spent in choosing fig sex increases the risks of reproductive failure^22^. Another possible explanation is the “no preference” hypothesis. Grafen and Godfray^15^, discussing vicarious selection in the dioecious fig–pollinator system, suggested that pollinating wasps cannot choose between male and female figs because there are no detectable differences between them. In fact, female trees need to attract wasps to produce seeds and male trees need to attract pollinators whose offspring will disperse their pollen, i.e. whose offspring will be attracted by female trees. Thus, trees of both sexes are strongly selected to mimic each other. Since at the receptive stage there are no obvious visual differences (in diameter, colour, etc.) between male and female figs and since chemical signals have been shown to be sufficient to attract specific pollinating fig wasps in the absence of other cues^13^, pollination by deceit in dioecious figs can be hypothesized to depend on mimicry in the chemical composition of floral scent.

Yet, the importance of chemical mimicry in maintaining a mutualism based on pollination by deceit in dioecious fig species is still largely unresolved. Previous studies seemed to indicate that different species of *Ficus* present alternative strategies in intersexual chemical mimicry, whether for pollination or for seed dispersal^23^,^26^,^27^,^28^. We hypothesize that in fig species where male and female trees flower synchronously, selection will favour a strong resemblance of the chemical signal emitted by figs of the two sexes in order to ensure that pollinators visit female figs. In contrast, in fig species where males and females flower mainly asynchronously, this selection among fig sexes could be relaxed (as female figs will be visited by wasps that do not have a choice between sexes). However, scent divergence between sexes would still be limited because figs are still under strong selection to produce odours allowing host species recognition.

In fig species, dioecy is a derived trait and has evolved at least twice from monoecy^29^; among the six subgenera of figs, dioecy occurs in three (*Ficus, Sycomorus* and *Sycidium)*. In the present study, using a large data set (seven fig species from the three dioecious subgenera, Table 1), we investigated the volatile profiles (composition and quantity) emitted by receptive figs of both sexes. We verified the role of these flower scents in host species-specific recognition and in ensuring constancy of pollinators. Comparing fig species in which males and females flower synchronously and asynchronously, we specifically examined the potential of volatile chemical signals in mediating intersexual mimicry in dioecious nursery pollination mutualisms by estimating the degree of intersexual chemical similarity in species with varied flowering phenologies.

## Results

### Specificity of the signal

**Figure 1:**
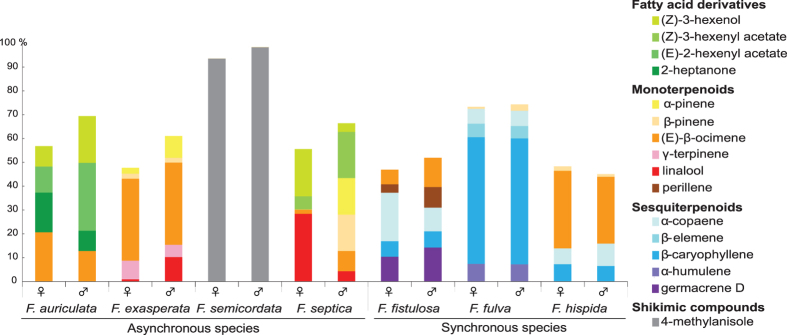
Proportions of the main compounds (representing more than 5% of the total scent) in the volatile bouquet emitted at receptivity by the two sexes of synchronous species (male and female figs flowering at the same time) and asynchronous species (male and female figs flowering at different times).

We identified a total of about 150 VOCs produced by the receptive figs of the seven dioecious species. The floral scents emitted by these figs were mainly composed of monoterpenes and sesquiterpenes. The blend of compounds was generally dominated by one or a few common terpenoid compounds, the identity of which differed among the species (Fig. 1); for example, β-caryophyllene for *F. fulva*, germacrene-D for *F. fistulosa*, or (E)-β-ocimene for *F. hispida* (see Supplementary Table S1 for more details). In one species, a rare compound dominated, i.e., 4-methylanisole in the scent of *F. semicordata*.

**Figure 2:**
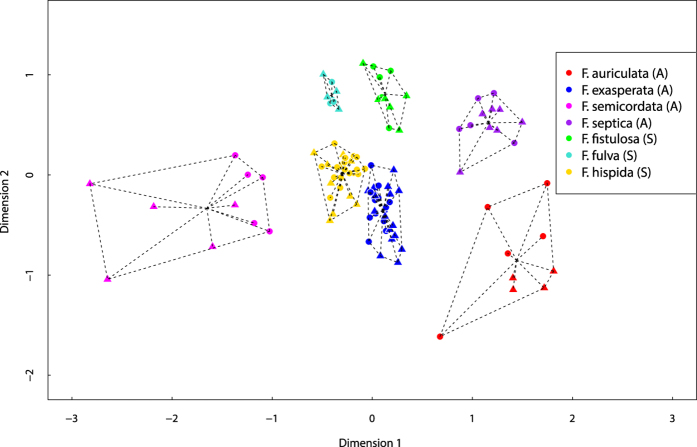
Non-metric multidimensional scaling (NMDS) ordination of chemical composition of all studied species at receptive stage, based on Bray-Curtis distance, rotated by principal component analysis (triangle: male; circle: female) (S: synchronous species, A: asynchronous species).

Scents of individuals from the same species are clearly grouped together based on the ordination (NMDS) of the odour samples from the different species (based on the Bray-Curtis distances) (Fig. 2). Moreover, the PERMANOVA confirmed that the volatile chemical profiles of the receptive figs varied significantly among the different species (see Table 2).

### Intersexual variation of scents

**Figure 3:**
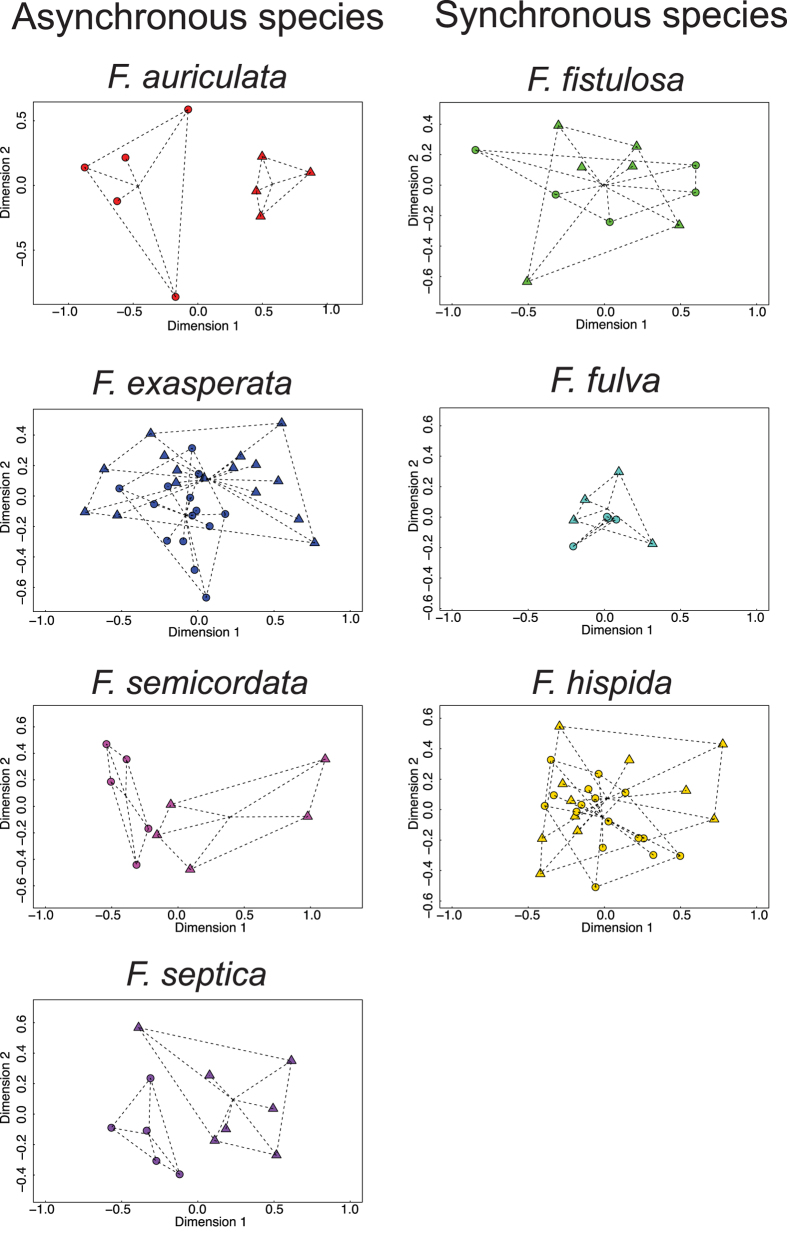
Non-metric multidimensional scaling (NMDS) ordination of chemical composition of the two sexes of all studied species at receptive stage, based on Bray-Curtis distance, rotated by principal component analysis (triangle: male; circle: female).

We then separated the data by species to test for the effect of sex on the volatile bouquet of VOCs emitted by each species. The species fell into two groups in these analyses (NMDS ordinations presented in Fig. 3). First, the volatile profiles of the sexes cannot be separated in species in which both sexes flower synchronously (namely *F. fistulosa, F. fulva*, and *F. hispida*). These results are confirmed by the PERMANOVAs, in which the factor ‘sex’ is not significant (p > 0.105, see Table 2). The second group consists of species in which the two sexes do not flower synchronously. For this group, volatile profiles showed significant variation between sexes. On the ordination graphs based on Bray-Curtis distances, VOCs emitted by receptive male and female figs are separated in three of the four asynchronous species, *F. auriculata, F. semicordata* and *F. septica* (Fig. 3). The PERMANOVA (for all species p ≤ 0.010, Table 2) shows a clear and significant effect of sex on the composition of the floral blends emitted by receptive figs of asynchronously flowering figs. For *F. exasperata*, even if the volatile profiles of male and female figs overlap on the ordination graphs (Fig. 3), the positions of the centroids of the groups are significantly separated statistically (Table 2).

For the species in which VOC composition significantly differed between sexes, we conducted a one-way Simper analysis to identify the compounds that best explain dissimilarities between sexes. In some species, this analysis revealed that major compounds were responsible for these dissimilarities between the bouquets of male and female figs, as in the case of *F. semicordata*, where the proportion of 4-methylanisole mainly explains this difference (18% of the variation between sexes). For the other species, this difference was explained not only by some major compounds but also by minor compounds such as decanal (5%), (Z)-3-hexenol, ethyl hexanoate, linalool and β-caryophyllene (all about 4%) for *F. auriculata*, or linalool, phenylethanol, (Z)-3-hexenol, hexyl acetate, (E)-2-hexenyl acetate, β-bourbonene and eugenol (all at about 3%) for *F. septica.* Finally, for *F. exasperata* differences between sexes in VOCs emitted by receptive figs were mainly due to a suite of compounds (both major and minor), although each only contributed to a small proportion (about 2%) of the dissimilarities. In *F. exasperata*, these compounds included methyl-acetophenone, linalool, methyl salicylate, terpinolene, (E)-β-ocimene, hexadecane, myrcene, perillene and α-copaene.

### Variation between sexes in quantity of volatile compounds emitted

**Figure 4:**
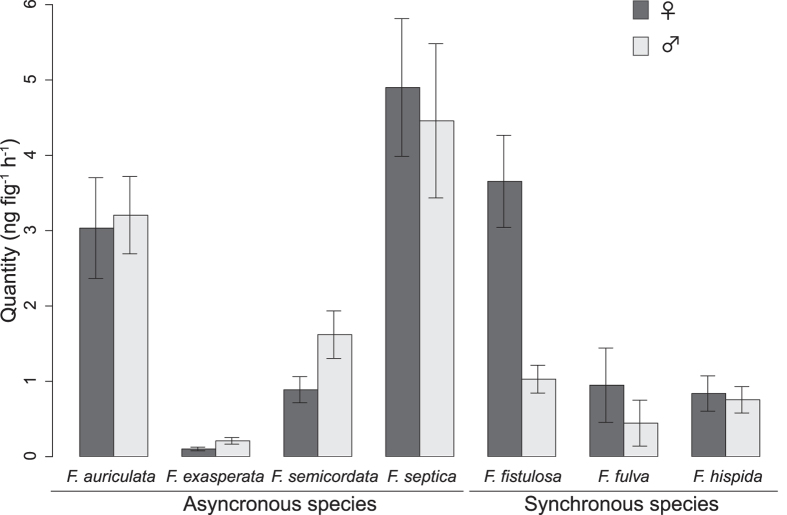
Mean quantity of VOCs (ng fig^−1^. hr^−1^) emitted by the different *Ficus* species and sexes.

Except for one species, *F. fistulosa*, there was no significant difference in the quantity of VOCs emitted by male and female figs during receptivity (Fig. 4, Table 3). For *F. fistulosa*, receptive female figs produced about four times more VOCs (total quantity) than did male figs (3.7 ± 1.4 ng fig^−1^h^−1^ for female figs versus 1.0 ± 0.45 ng fig^−1^h^−1^ for male figs), although figs of the sexes are equal in size (see mean diameter in Table 1). Among the different species, however, fig diameter (Table 1) is a parameter that contributes to explain differences in the amount of VOCs produced, and not surprisingly species with larger figs, such as *F. auriculata* and *F. septica*, produced higher quantities of volatile compounds.

## Discussion

Our results represent to the best of our knowledge the first evidence for a strong effect of flowering phenology as a selective pressure on intersexual chemical mimicry. They also reinforce previous results on interspecies variation of flower scent in *Ficus*^30^. Indeed, they highlight the importance of flower scents as a crucial trait for avoiding host identification mistakes in *Ficus* species and maintaining highly specific interactions in these and other horizontally transmitted mutualisms^13^.

Our findings corroborate the role of flower scents as a pre-zygotic barrier between closely related plant species in nursery pollination systems^31^, which could lead to reproductive isolation during speciation^32^. Moreover, our results confirm that neither potential interpopulation variation nor intersexual variation affects this clear interspecies variation of flower scent in dioecious *Ficus* species. Furthermore, behavioural experiments conducted on the pollinators of some of the species we studied (*F. hispida, F. semicordata*, and *F. carica*) have already shown that pollinating fig wasps use this specific chemical message as a cue to locate their mutualistic host^33^ (M. Proffit & C. Soler, unpubl. data). Once again, the “endless” variation in the composition of floral signals allows fig species to elaborate quite complex and specific blends in order to attract their specific pollinators^13^.

Regarding intersexual mimicry in dioecious species, our results provide evidence within a similar phylogenetic background that there are two different answers to the question of ‘how to be a dioecious fig species’ that depend on flowering phenology. On one hand, in dioecious species in which male and female trees flower synchronously, fig-pollinating wasps loaded with pollen from their natal male figs have a simultaneous choice between receptive male and female figs within the same population. For these synchronous species, it is important to ensure that female pollinating wasps cannot discriminate between sexes in order to maintain both male (pollen and pollinating wasp production) and female (seed production) functions. Consequently, this leads to tighter intersexual mimicry in flower scents, as we have shown for three synchronously flowering dioecious species belonging to two different *Ficus* subgenera, *F. fulva*, *F. fistulosa* and *F. hispida*.

In only one of these synchronously flowering species did we find a difference in the quantity of compounds released by receptive figs, with the deceptive sex (female figs) producing greater quantities of VOCs. This may be consistent with the need to ensure pollinator visitation even to the fatally deceptive female sex. In other less exclusive but still specialized interactions, and in those without the fatal consequences of choosing the wrong sex, such as the mutualistic interactions between the seed-predating and pollinating noctuid moth *Hadena bicruris* and its dioecious host *Silene latifolia*, Dötterl *et al.*^34^ also found no difference in the quality and quantity of VOCs emitted by female and male flowers (but see^35^). In *Salix caprea*, an insect-pollinated dioecious species, total scent emission was higher in male flowers, and some sex-specific differences in relative scent composition were detected. However, naïve honey bees were equally attracted to the scent of both sexes^36^. Remarkable qualitative intersexual differences in floral scents were also found by Okamoto and collaborators^37^ in their detailed study of sexual differences in floral scent of the monoecious species of the tribe Phyllantheae (Malpighiales, Phyllanthaceae) that are pollinated by *Epicephala* moths (Lepidoptera, Gracillariidae) in interactions that include some examples of nursery pollination mutualisms. In these examples, differences in scents exist between male and female flowers of the same individual, highlighting the possibility for sexual scent divergence even at the intra-individual level. In addition, these authors show that mated female *Epicephala* moths preferred the scent of male flowers over that of female flowers. In contrast to the interactions between figs and their pollinating wasps, in the nursery pollination interaction in Phyllanthaceae, it is mandatory for the pollinators to visit first male, then female flowers in order to gain the reward provided by the interaction. Even though the mechanism triggering the visit of female flowers following that of male flowers has not been elucidated in the Phyllanthaceae system, sexual dimorphism of floral scent and its discrimination by pollinators have been interpreted as adaptive for both plant and pollinator^37^.

On the other hand, for fig species in which individuals of the two sexes flower at different times of the year, the short-lived pollinating wasps have no choice and simply enter the available receptive figs within the population based on the host-specific chemical signal. In such situations, the selective pressure on intersexual mimicry should be relaxed. In fact, intersexual variation of floral scents is detectable at the intraspecific level for the species of this category that we studied. However, this intraspecific variation between sexes in flower scents should be less marked than the interspecific variation that allows pollinators to distinguish signals from the specific host fig species to avoid mistakes, especially if fig species occur in sympatry^38^, as for *F. hispida* and *F. exasperata* in our study. Our results thus suggest that this sexual dimorphism is not likely to disrupt the species-specific encounter of the two partners, confirming the strong species isolation mechanisms expected in dioecious figs^38^.

The sexual difference in dioecious figs appears to rely on VOC identity rather than on the overall VOC quantity, in contrast to most previously studied angiosperm species, where intersexual differences are mostly due to a higher total quantity of VOCs emitted by male flowers^5^ (but see^39^). In another nursery pollination mutualism characterized by partial asynchrony in the flowering of male and female plants, that between *Chamaerops humilis*, the dioecious dwarf palm, and *Derelomus chamaeropsis*, its pollinating weevil, male plants produced four times more VOCs than female plants. However, the qualitative composition of scents did not differ between the sexes, allowing female plants, the deceptive sex, to also attract the species-specific pollinating weevil^40^. These findings suggest that quality and quantity can interact in diverse ways based on particular ecologies and contexts and that no one unique prediction is possible.

For the dioecious fig species in which males and females flower asynchronously, differences in the VOCs emitted may be accounted for by differences in the proportions of some major compounds, such as 4-methylanisole for *F. semicordata*. However, some minor compounds from different pathways may also play a role in these intersexual differences of VOC bouquets, such as methyl-acetophenone, linalool, methyl salicylate or α-copaene for *F. exasperata*. The statistical analysis used in the present study, performed as recommended in recent studies comparing flower scents^36^,^37^,^41^, minimizes the potential effect of major compounds on their contribution to the total variance.

This study on several fig species provides novel insights into our understanding of the occurrence of intersexual chemical mimicry in dioecious species. In her well-documented review, Ashman^5^ reported that the occurrence of sexual dimorphism is usually explained in the literature by i) sexual selection, ii) allometric relations, iii) honest signals, iv) directed pollinator movement, or v) post-pollination partners. In contrast, the absence of sexual dimorphism may be explained by intersexual mimicry or by between-sex genetic constraints. These various explanations have come from different studies conducted on different species, with varied evolutionary and ecological histories, and cannot really be applied in our *Ficus* case. In dioecious figs we found intersexual mimicry in species with synchronous flowering between the sexes and relaxed intersexual mimicry in species where male and female fig availability is temporally separated. First, given the special biology of dioecious figs, there is no advantage via sexual selection accruing to males that have scent profiles more attractive to pollinators than those of females, since visits (albeit fatal) to female figs (in which seeds are produced) by pollinators carrying its pollen are necessary for the function of a male tree to be performed. Second, for all the fig species we studied, male and female figs have the same size, shape and colour, but they differ greatly in the rewards offered. Since this system is based on deception (in which female fig trees are the fatally deceptive sex), the issue of honesty of signals leading to sexual dimorphism^42^ does not arise. Furthermore, since pollinators have only one opportunity to enter a fig (either a true fig wasp nursery in male trees or a fatally attractive deceptive one in female trees), the question of directed pollinator movement from one sex to another by sexual dimorphism in floral scent also does not apply. The only remaining explanation from Ashman’s review^5^ is that which takes into account post-pollination visitors. These are effectively different for the two sexes: male figs are parasitized by other wasps, contrary to most female figs^43^. However, this sexual difference in attraction of post-pollination parasites holds not only for asynchronous species of dioecious figs but also for synchronous species: since no sexual variation of scents has been detected in the latter case, it is unlikely that any explanation based on selective pressures exerted by post-pollination interactions will apply here. This therefore means in figs that there is no advantage to sexual dimorphism in floral scent via any of the selection pressures often associated with scent dimorphism^5^. The scent of the fatally attractive female must converge towards that of the rewarding male sex in figs, which may imply that females must mimic males, or alternatively that floral scent in each sex must show directional selection towards the other in order for individual trees to achieve fitness and consequently for the mutualism to survive. This is especially true when both sexes are simultaneously available to the pollinator. However, when the sexes are not simultaneously available, as in asynchronous species, it is possible that the host-specific chemical signal (which is likely a blend of volatiles) remains constant between the sexes, but that additional VOCs—that are not sufficient to mask or blur the species-specific host-recognition signal for the pollinator—are unconstrained to vary, resulting in a statistical difference between the scents of the sexes. So far, for these species we do not know the specific compounds in fig scents that are responsible for pollinator attraction. However, we do have some evidence that pollinators of several *Ficus* species are able to detect not only the major compounds emitted by receptive figs but also some minor compounds^44^ (L. Zongbo *et al.* and M. Proffit *et al.*, unpublished data). Therefore, the possible effect on behaviour (e. g. attraction or repulsion) of these minor compounds should not be neglected in fig/wasp interactions, a precaution that echoes those suggested for other plant–insect interactions^45^. Consequently, thorough analysis of the contribution of the different compounds should be complemented by experimental behavioural studies, or by GC/EAD (gas chromatography/electro-antennographic detection), in order to know precisely which compounds are active in attraction^24^,^46^.

We have thus shown in this study the existence of two alternative ways to be a dioecious fig species that depend on differences in the synchrony of flowering between sexes. Since dioecious *Ficus* are in fact anatomically gynodioecious, female and male figs are structurally not identical. Observing intersexual variations of scents (as occur in asynchronous species) is thus not surprising and may be explained by different scent-emitting structures. However, this constitutive difference is thwarted by vicarious selection for intersexual chemical mimicry^15^, to prevent the breakdown of the mutualism, in the case of synchronously flowering species. Nevertheless, in both cases, the deceptive sex is pollinated, and the interactions between pollinating fig wasps and dioecious fig species are successfully maintained.

## Material and Methods

### Studied species

In order to have a good phylogenetic and geographical representation for this comparative study, we chose species with different flowering phenologies (synchronous *versus* asynchronous flowering of males and females) from different sections among the three subgenera of dioecious species, and from different geographical regions distributed over tropical, subtropical regions of the Old World (Table 1). No dioecious fig species occur in the New World. From published data or our personal observations, we can divide these species into two groups regarding their flowering phenology^47^,^48^. In the first group, the two sexes flower in synchrony in our studied populations: *F. hispida, F. fistulosa* and *F. fulva*; in the second one, flowering phenology is mainly asynchronous, with receptive figs of male and female trees co-occurring very rarely in time: *F. auriculata*, *F. exasperata*, *F. semicordata* and *F. septica*^27^,^28^,^33^.

### Collection of VOCs

Floral scents were collected only when it was clear that the selected figs had reached receptivity, i.e. when they attract their pollinators. This stage is easily recognizable in figs since the ostiole ( = opening of the fig) is soft, bracts lining this ostiolar opening are not tightly adpressed, and many pollinators fly around the receptive figs.

The scents were collected by the adsorption-desorption headspace technique^17^. For odour collection, only receptive figs were collected from the tree and directly enclosed in polyethylene terephthalate (Nalophan®) bags, which have been shown not to release or adsorb volatiles. For these extractions we used two different methods, depending on the type of volatile collection traps:

1. For traps containing 30 mg of Alltech Super Q adsorbent (ARS Inc., Gainesville, FL, USA), airflow was maintained through the bags by two pumps. Entrance and exit flow rates (controlled by flow meters) were 400 and 300 ml min^-1^. The extraction duration was 3 h. Trapped volatiles were eluted with 150 μl of dichloromethane, and two internal standards (nonane and dodecane, at 200 ng μl^−1^ for all species except *F. semicordata*, where octane, 117.00 ng μl^−1^, and decyl acetate, 144.17 ng μl^−1^, were used because dodecane was present in the VOCs released by this species) were added to each sample for gas chromatography. VOCs were analysed by injection in a CP-3800 (Varian Inc., Palo Alto, CA, USA) gas chromatograph with a flame ionization detector (FID) coupled with a Saturn 2000 mass spectrometer (Varian Inc., Palo Alto, USA).
2. For traps filled with 3 mg of a 1:1 mixture of Tenax-TA 60–80 and Carbotrap 20–40 (containing a 110 ng μl^−1^ solution of nonane and dodecane as internal standards), air was drawn out of the bags and over the trap for 5 min by a pump with controlled airflow (200 ml min^−1^) after keeping the figs and traps in the bags for 30 min. In this thermal-desorption method, the samples were analysed on the same CP-3800 Varian GC/MS using a 1079 injector fitting to the ChromatoProbe® device.

For both techniques we always performed blank extractions with empty bags to control for contaminant compounds. Odour collection was always performed under natural light and at ambient temperatures between 12:00 and 15:00 hours, corresponding to the insects’ period of maximum activity during our field season. Comparisons of the efficiency of the two types of traps (M. Proffit, unpubl. data) allowed us to use both types of volatile collections in this comparative study. Moreover, we always used the same technique to collect the volatiles of one species and straightforward comparison of scents emitted by the two sexes was thus always possible.

The relative proportions of the compounds emitted by figs were determined for each sample using GC-FID chromatograms. In addition, for each sample, we estimated the total quantities of volatile compounds by using the peak areas of both internal standards in GC-FID chromatograms as a reference quantity. Peak areas of all the compounds were determined, and then divided by the peak areas of the standards and multiplied by the known amounts for the standards.

### Analysis of VOCs

Traps were stored in the dark in a freezer until analysis. For both sampling techniques a Varian column CP-SIL low blend MS (30 m, ID 0.25 mm, film thickness 0.25 μm) and helium as carrier gas (at 1 ml min^−1^) were used. The injector split vent was opened at a ratio of 1:4. Oven temperature was programmed at 50 °C during 3 min; then increasing by 3 °C min^–1^ to 100 °C, by 2.7 °C min^–1^ to 140 °C, by 2.4 °C min^–1^ to 180 °C and by 6 °C min^–1^ to 250 °C. For Chromatoprobe® samples, the injection was done at 40 °C for 1 min, followed by a warm-up for 3 min, until 250 °C, at the rate of 200 °C min^–1^. The temperature program for the analysis was: 40 °C for 3 min, ramped from 40 °C to 100 °C at 3.3 °C min^–1^ to 250 °C, from 100 °C to 140 °C at 2.9 °C min^–1^, then 140 °C to 180 °C at 2.7 °C min^–1^, and finally from 180 °C to 250 °C at 10 °C min^–1^. Temperature was maintained at 250 °C for 8 subsequent minutes.

Compound identification was based on computer matching of the mass spectra with NIST 98 MS library, on retention indices reported in the literature^49^ and, whenever available, by injection of reference compounds. By comparing spectra of each sample with the respective control sample (empty bag, same day and conditions of collection), putative contaminant compounds were subtracted.

### Data analysis

All statistical analyses were performed using R^50^. In order to compare patterns of scent composition both among different species and between sexes within each species, we performed a non-metric multidimensional scaling (NMDS) using the function meta MDS in the package Vegan^51^. For the comparison of volatile profiles among different *Ficus* species we used the relative proportions of all the compounds emitted by the seven species, whereas for inter-sexual variation of scent each species was analysed separately. Prior to the analysis, data were square-root transformed and then standardized using a Wisconsin double standardization. A data matrix of pairwise comparisons among samples was then calculated using the Bray-Curtis distance index, which ranges between 0 and 1^52^. NMDS was used to find the best low-dimensional representation of the distance matrix. Finally, the solution was scaled by rotating it, so that the largest variance of samples was on the first axis^51^,^53^. The null hypothesis of no difference in patterns of scent composition among species and between sexes for each species was tested with a permutational multivariate analysis of variance (PERMANOVA) using the function ‘adonis’ in Vegan. PERMANOVA is a nonparametric (in the case of one-factor models) permutation-based version of the multivariate analysis of variance, and thus with no assumption regarding the distributions of the original variables^54^. However, PERMANOVAs are very sensitive to heteroscedasticity^55^. Therefore, prior to analysis homoscedasticity was confirmed using a multivariate analogue of Levene’s test for homogeneity of variance. PERMANOVAs were run on the Bray-Curtis distance index with 1000 permutations per analysis. A similarity percentage (one-way SIMPER, with factor sex) was used to identify the compounds that best explained dissimilarities (up to 30%) between sexes.

For each species, we used a linear model to test if there were significant effects of sex on the total quantities of VOCs emitted by figs. The goodness-of-fit of all models was evaluated by inspecting the models’ residuals; the normality of the residuals was tested using a Shapiro-Wilk’s test. For data with residuals not normally distributed, a log transformation was used to ensure their normality. Significance (p < 0.05) was assessed by testing the change in deviance after the removal of the factor sex from the model.

## Additional Information

**How to cite this article**: Hossaert-McKey, M. *et al.* How to be a dioecious fig: Chemical mimicry between sexes matters only when both sexes flower synchronously. *Sci. Rep.* **6**, 21236; doi: 10.1038/srep21236 (2016).

## Supplementary Material

Supplementary Information

## Figures and Tables

**Table 1:** The studied species, their provenances, their flowering phenology and the diameter of their figs.

Species	Subgenus	Section	Sites	Geographical coordinates	Fig presentation	Phenology	Number of trees sampled		Mean and SD of fig diameters (mm)	
							Male	Female	Male	Female
*F. fistulosa*	*Sycomorus*	*Sycocarpus*	Bruneï	4°94′N, 114.95′E	Cauliflory	synchronous	6	5	11.9 ± 1.5	12.9 ± 1.7
*F. fulva*	*Ficus*	*Ficus*	Bruneï	4°94′N, 114°95′E	Ramiflory	synchronous	4	3	—	—
*F. hispida*	*Sycomorus*	*Sycocarpus*	Karnataka (India)	13°51′N, 75°09′E	Cauliflory	synchronous	8	10	15.2 ± 2.1	14.9 ± 2.5
*F. auriculata*	*Sycomorus*	*Sycomorus*	Yunnan (China)	21°41′N, 101°25′E	Figs on stolons	asynchronous	4	4	36.4 ± 5.2	34.9 ± 7.1
*F. exasperata*	*Sycidium*	*Sycidium*	Karnataka (India)	13°51′N, 75°09′E	Ramiflory	asynchronous	10	13	10.5 ± 2.1	10.3 ± 1.9
*F. semicordata*	*Sycomorus*	*Hemicardia*	Yunnan (China)	21°41′N, 101°25′E	Figs on stolons	asynchronous	5	5	16.3 ± 0.3	12.2 ± 0.2
*F. septica*	*Sycomorus*	*Sycocarpus*	Taiwan	25°10′N, 121°30′E	Ramiflory	asynchronous	7	5	15.4 ± 0.3	15.9 ± 1.1

**Table 2:** Global results on interspecies variations, and on comparison between sexes of the proportion of volatile compounds emitted by figs at receptivity within each species.

Effect		Stress in %	Df	F	p
Species	Global	18.43	6, 106	20.75	**0.001**
Sex	*F. fistulosa* (S)	11.44	1, 10	0.72	0.660
Sex	*F. fulva* (S)	6.576	1, 7	0.88	0.740
Sex	*F. hispida* (S)	17.29	1, 25	1.51	0.105
Sex	*F. auriculata* (A)	9.04	1, 8	3.90	**0.007**
Sex	*F. exasperata* (A)	16.37	1, 29	2.80	**0.001**
Sex	*F. semicordata* (A)	5.84	1, 9	2.36	**0.042**
Sex	*F. septica* (A)	13.10	1, 11	2.59	**0.010**

Permutational multivariate analysis of variance (PERMANOVA) performed on proportion of VOCs (transformation first square-root then Wisconsin double standardization). Significant *P*-values (p < 0.05) are indicated in bold. (S): Synchronous species, (A): Asynchronous species.

**Table 3:** Comparison between sexes of the total amount of VOCs emitted by receptive figs.

Species	Transformation	Df	*F*	p
*F. fistulosa* (S)	no	1, 10	19.924	**0.002**
*F. fulva* (S)	no	1, 5	0.294	0.617
*F. hispida* (S)	log	1, 27	0.259	0.615
*F. auriculata* (A)	no	1, 8	0.094	0.851
*F. exasperata* (A)	log	1, 29	1.483	0.233
*F. semicordata* (A)	no	1, 9	4.115	0.077
*F. septica* (A)	no	1, 11	0.004	0.765

Tested using linear model and analysis of deviance on the quantity of volatile compounds (log transformation when needed). Significant *P*-values (p < 0.05) are indicated in bold. (S): Synchronous species, (A): Asynchronous species.

## References

[b1] Charlesworth D. Theories of the evolution of dioecy. Gender and sexual dimorphism in flowering plants (eds Geber M. A., Dawson T. E., & Delph L. F. ) 33–60 (Springer, Berlin, 1999).

[b2] Renner S. S. & Ricklefs R. E. Dioecy and its correlates in the flowering plants. Am. J. Bot. 82, 596–606 (1995).

[b3] Barrett S. C. H. The evolution of plant reproductive systems: how often are transitions irreversible? Proc. R. Soc. Lond. B. 280, 20130913 (2013). 10.1098/rspb.2013.0913 PMC3712442 23825207

[b4] Vamosi J. C. & Vamosi S. M. The role of diversification in causing the correlates of dioecy. Evolution 58, 723–731 (2004). 15154548 10.1111/j.0014-3820.2004.tb00405.x

[b5] Ashman T. L. Sniffing out patterns of sexual dimorphism in floral scent. Funct. Ecol. 23, 852–862 (2009).

[b6] Renner S. S. Rewardless flowers in the Angiosperms and the role of insect cognition in their evolution. Plant-pollinator interactions from specialization to generalization (eds Waser N.M. & Ollerton J. ) 123–144 (The University of Chicago Press, Chicago, Illinois, 2006).

[b7] Jersáková J., Johnson S. D. & Kindlmann P. Mechanisms and evolution of deceptive pollination in orchids. Biol. Rev. 81, 219–235 (2006). 16677433 10.1017/S1464793105006986

[b8] Raguso R. A. Wake up and smell the roses: the ecology and evolution of floral scent. Annu. Rev. Ecol. Evol. Syst. 39, 549–569 (2008).

[b9] Steiner K. E., Kaiser R. & Dötterl S. Strong phylogenetic effects on floral scent variation of oil-secreting orchids in South Africa. Am. J. Bot. 98, 1663–1679 (2011). 21965135 10.3732/ajb.1100141

[b10] Knudsen J. T. & Gershenzon J. The chemical diversity of floral scent. Biology of floral scent (eds Natalia Dudareva & Eran Pichersky) 27–52 (Taylor and Francis, CRC press, Boca Raton, 2006).

[b11] Parachnowitsch A. L. & Manson J. S. The chemical ecology of plant-pollinator interactions: recent advances and future directions. Curr. Opin. Insect Sci. 8, 41–46 (2015). 10.1016/j.cois.2015.02.005 32846674

[b12] Parachnowitsch A. L., Raguso R. A. & Kessler A. Phenotypic selection to increase floral scent emission, but not flower size or colour in bee-pollinated *Penstemon digitalis*. New Phytol. 195, 667–675 (2012). 22646058 10.1111/j.1469-8137.2012.04188.x

[b13] Hossaert-McKey M., Soler C., Schatz B. & Proffit M. Floral scents: their roles in nursery pollination mutualisms. Chemoecology 20, 75–88 (2010).

[b14] Dufaÿ M. & Anstett M. Conflicts between plants and pollinators that reproduce within inflorescences: evolutionary variations on a theme. Oikos 100, 3–14 (2003).

[b15] Grafen A. & Godfray H. C. J. Vicarious selection explains some paradoxes in dioecious fig pollinator systems. Proc. R. Soc. Lond. B. 245, 73–76 (1991).

[b16] Janzen D. H. How to be a fig. Annu. Rev. Ecol. Syst. 10, 13–51 (1979).

[b17] Grison-Pigé L., Bessière J.-M. & Hossaert-McKey M. Specific attraction of fig-pollinating wasps: Role of volatile compounds released by tropical figs. J. Chem. Ecol. 28, 283–295 (2002). 11925068 10.1023/a:1017930023741

[b18] Hossaert-McKey M., Gibernau M. & Frey J. E. Chemosensory attraction of fig wasps to substances produced by receptive figs. Entomol. Exp. Appl. 70, 185–191 (1994).

[b19] Ware A. B., Kaye P. T., Compton S. G. & van Noort S. Fig volatiles—Their role in attracting pollinators and maintaining pollinator specificity. Plant Syst. Evol. 186, 147–156 (1993).

[b20] Kjellberg F., Gouyon P., Ibrahim M., Raymond M. & Valdeyron G. The stability of the symbiosis between dioecious figs and their pollinators: a study of *Ficus carica* L. and *Blastophaga psenes* L. Evolution 41, 693–704 (1987). 10.1111/j.1558-5646.1987.tb05846.x 28564365

[b21] Verkerke W. Structure and function of the fig. Experientia 45, 612–622 (1989).

[b22] Patel A., Anstett M., Hossaert McKey M. & Kjellberg F. Pollinators entering female dioecious figs: why commit suicide? J. Evol. Biol. 8, 301–313 (1995).

[b23] Grison-Pigé L. *et al.* Limited intersex mimicry of floral odour in *Ficus carica*. Funct. Ecol. 15, 551–558 (2001).

[b24] Soler C. C., Proffit M., Bessière J.-M., Hossaert-McKey M. & Schatz B. Evidence for intersexual chemical mimicry in a dioecious plant. Ecol. Lett. 15, 978–985 (2012). 22762353 10.1111/j.1461-0248.2012.01818.x

[b25] Kjellberg F., Doumesche B. & Bronstein J. L. Longevity of a fig wasp (*Blastophaga psenes*). P. K. Ned. Akad. C Biol. 91, 117–122 (1988).

[b26] Chen C. & Song Q. Responses of the pollinating wasp *Ceratosolen solmsi marchali* to odor variation between two floral stages of *Ficus hispida*. J. Chem. Ecol. 34, 1536–1544 (2008). 19015920 10.1007/s10886-008-9558-4

[b27] Patel A. Phenological patterns of *Ficus* in relation to other forest trees in southern India. J. Trop. Ecol. 13, 681–695 (1997).

[b28] Borges R. M., Bessiere J. -M. & Hossaert-McKey M. The chemical ecology of seed dispersal in monoecious and dioecious figs. Funct. Ecol. 22, 484–493 (2008).

[b29] Jousselin E., Rasplus J. & Kjellberg F. Convergence and coevolution in a mutualism: evidence from a molecular phylogeny of *Ficus*. Evolution 57, 1255–1269 (2003). 12894934 10.1554/02-445

[b30] Grison L., Edwards A. A. & Hossaert-McKey M. Interspecies variation in floral fragrances emitted by tropical *Ficus* species. Phytochemistry 52, 1293–1299 (1999).

[b31] Friberg M., Schwind C., Raguso R. A. & Thompson J. N. Extreme divergence in floral scent among woodland star species (*Lithophragma* spp.) pollinated by floral parasites. Ann. Bot. 111, 539–550 (2013). 23365407 10.1093/aob/mct007 PMC3605946

[b32] Schiestl F. P. Ecology and evolution of floral volatile-mediated information transfer in plants. New Phytol. 206, 571–577 (2015). 25605223 10.1111/nph.13243

[b33] Chen C. *et al.* Private channel: a single unusual compound assures specific pollinator attraction in *Ficus semicordata*. Funct. Ecol. 23, 941–950 (2009).

[b34] Dötterl S., Wolfe L. M. & Jürgens A. Qualitative and quantitative analyses of flower scent in *Silene latifolia*. Phytochemistry 66, 203–213 (2005). 15652577 10.1016/j.phytochem.2004.12.002

[b35] Waelti M. O., Page P. A., Widmer A. & Schiestl F. P. How to be an attractive male: floral dimorphism and attractiveness to pollinators in a dioecious plant. BMC Evol. Biol. 9, 190 (2009). 19660122 10.1186/1471-2148-9-190 PMC2738674

[b36] Dötterl S., Glück U., Jürgens A., Woodring J. & Aas G. Floral reward, advertisement and attractiveness to honey bees in dioecious *Salix caprea*. PLoS One 9, 10.1371/journal.pone.0093421 (2014). PMC3968154 24676333

[b37] Okamoto T., Kawakita A., Goto R., Svensson G. P. & Kato M. Active pollination favours sexual dimorphism in floral scent. Proc. R. Soc. Lond. B. 280, 20132280 (2013). 10.1098/rspb.2013.2280 PMC3813343 24266037

[b38] Yang L. Y. *et al.* The incidence and pattern of copollinator diversification in dioecious and monoecious figs. Evolution 69, 294–304 (2014). 25495152 10.1111/evo.12584 PMC4328460

[b39] Ashman T. L., Bradburn M., Cole D. H., Blaney B. H. & Raguso R. A. The scent of a male: the role of floral volatiles in pollination of a gender dimorphic plant. Ecology 86, 2099–2105 (2005).

[b40] Dufaÿ M. & Anstett M. C. Cheating is not always punished: killer female plants and pollination by deceit in the dwarf palm *Chamaerops humilis*. J. Evol. Biol. 17, 862–868 (2004). 15271086 10.1111/j.1420-9101.2004.00714.x

[b41] Proffit M. *et al.* Can chemical signals, responsible for mutualistic partner encounter, promote the specific exploitation of nursery pollination mutualisms ? The case of figs and fig wasps. Entomol. Exp. Appl. 131, 46–57 (2009).

[b42] Hemborg Å. M. & Bond W. J. Different rewards in female and male flowers can explain the evolution of sexual dimorphism in plants. Biol. J. Linn. Soc. 85, 97–109 (2005).

[b43] Borges R. M. How to be a fig wasp parasite on the fig–fig wasp mutualism. Curr. Opin. Insect Sci. 8, 34–40 (2015). 10.1016/j.cois.2015.01.011 32846670

[b44] Wang G., Compton S. G. & Chen J. The mechanism of pollinator specificity between two sympatric fig varieties: a combination of olfactory signals and contact cues. Ann. Bot. 111, 173–181 (2013). 23179860 10.1093/aob/mcs250 PMC3555521

[b45] Clavijo Mccormick A., Gershenzon J. & Unsicker S. B. Little peaks with big effects: establishing the role of minor plant volatiles in plant–insect interactions. Plant Cell Environ. 37, 1836–1844 (2014). 24749758 10.1111/pce.12357

[b46] Borges R. M. On the air: broadcasting and reception of volatile messages in brood-site pollination mutualisms. Signaling and Communication in Plants (eds Blande J. & Glinwood R. ) in press (Springer, Berlin, 2016).

[b47] Kuaraksa C., Elliott S. & Hossaert-McKey M. The phenology of dioecious *Ficus* spp. tree species and its importance for forest restoration projects. For. Ecol. Manag. 265, 82–93 (2012).

[b48] Bain A. *et al.* Plasticity and diversity of the phenology of dioecious *Ficus* species in Taiwan. Acta Oecol. 57, 124–134 (2014).

[b49] Adams R. P. Identification of essential oil components by Gas Chromatography/Mass Spectroscopy, 4th edition. (Allured Publishing, Carol Stream, Illinois, USA, 2007).

[b50] R: A language and environment for statistical computing. R Foundation for Statistical Computing, V., Austria. URL http://www.R-project.org/. (R Core Team 2014).

[b51] Oksanen J. *et al.* Vegan: Community Ecology Package. R package version 2.0–7. (2013).

[b52] Bray J. & Curtis J. An ordination of the upland forest communities of southern Wisconsin. Ecol. Monogr. 27, 325–349 (1957).

[b53] Cornille A. *et al.* Floral volatiles, pollinator sharing and diversification in the fig-wasp mutualism: insights from *Ficus natalensis*, and its two wasp pollinators (South Africa). Proc. R. Soc. Lond. B. 279, 1731–1739 (2012). 10.1098/rspb.2011.1972 PMC3297447 22130605

[b54] Anderson M. J. A new method for non‐parametric multivariate analysis of variance. Austral Ecol. 26, 32–46 (2001).

[b55] Warton D. I., Wright S. T. & Wang Y. Distance‐based multivariate analyses confound location and dispersion effects. Method. Ecol. Evol. 3, 89–101 (2012).

