# Supplementary material for: How to be a dioecious fig: Chemical mimicry between sexes matters only when both sexes flower synchronously
Source: Sci Rep. 2016 Feb 18;6:21236. doi: 10.1038/srep21236 (PMC4758059; doi:10.1038/srep21236)
Supplement: Supplementary Information [file srep21236-s1.pdf]

Supplementary information:

Title of the manuscript: "How to be a dioecious fig: Chemical mimicry between sexes matters only when both sexes flower synchronously"

Authors: Hossaert-McKey M., Proffit M., Soler C.C.L., Chen C., Bessière J-M., Schatz B., and Borges R.M.

Supplementary Table S1 Volatile compounds emitted by receptive figs (mean percentages) for the seven fig species (both sexes). Mean ± SD; compounds > 5%; * VOCs for which identification was verified by injection of authentic standards).

**Compounds**	**Asynchronous species**							
	Ficus auriculata		***Ficus exasperata***		***Ficus semicordata***		***Ficus septica***	
	**Female**	**Male**	**Female**	**Male**	**Female**	**Male**	**Female**	**Male**
**Fatty acid derivatives**								
**2-heptanone**	16.70 ± 23.93	8.48 ± 9.63	-	-	-	-	3.20 ± 6.31	0.30 ± 0.60
**2-hexyl acetate**	0.75 ± 0.75	-	0.05 ± 0.18	0.14 ± 0.58	-	-	0.50 ± 0.78	1.84 ± 1.50
**tridecanol**	-	-	-	-	-	-	2.15 ± 4.13	0.22 ± 0.59
**undecanal**	-	-	-	-	-	-	1.55 ± 1.36	0.40 ± 0.67
**undecane ***	-	-	1.54 ± 2.70	0.64 ± 1.08	-	-	-	-
**nonanal ***	-	-	-	-	-	-	-	3.83 ± 6.46
**decanal ***	-	2.33 ± 2.26	-	-	-	-	0.33 ± 0.45	3.01 ± 4.06
**tridecane ***	-	0.10 ± 0.21	1.35 ± 2.67	0.17 ± 0.32	-	-	0.14 ± 0.17	0.73 ± 0.80
**tetradecane ***	-	-	0.96 ± 0.85	0.11 ± 0.21	0.05 ± 0.07	0.01 ± 0.01	1.11 ± 0.65	2.46 ± 2.23
**(Z)-3-hexenol**	8.60 ± 8.64	19.53 ± 3.69	-	-	-	-	19.78 ± 14.54	3.53 ± 4.88
**(E)-2-hexenyl acetate**	10.93 ± 4.92	28.53 ± 22.58	-	-	-	-	0.50 ± 0.78	1.84 ± 1.50
**(Z)-3-hexenyl acetate ***	-	-	1.60 ± 1.68	0.56 ± 0.68	-	-	5.52 ± 6.24	19.45 ± 23.69
**Monoterpenoids**								
**cis-linalool oxide (furanoid) ***	-	-	-	0.08 ± 0.31	-	-	-	-
**linalool ***	0.71 ± 1.59	7.26 ± 7.39	0.95 ± 0.83	10.30 ± 12.92	0.07 ± 0.12	-	28.39 ± 18.89	4.33 ± 5.27
**(*E*)-β-ocimene ***	20.65 ± 18.01	12.86 ± 12.46	34.36 ± 20.26	34.45 ± 19.62	1.14 ± 1.29	0.09 ± 0.10	1.78 ± 1.64	8.52 ± 16.80
**α-pinene ***	2.36 ± 5.29	6.01 ± 7.20	2.46 ± 1.03	9.18 ± 15.62	0.10 ± 0.11	0.02 ± 0.03	2.82 ± 2.84	2.90 ± 5.04
**β-pinene ***	-	-	2.13 ± 1.94	2.01 ± 2.53	0.02 ± 0.03	0.01 ± 0.01	0.06 ± 0.12	15.27 ± 25.26

**1.8-cineole ***	-	-	1.18 ± 1.71	1.17 ± 2.50	0.23 ± 0.52	-	-	0.39 ± 1.04
**perillene**	1.13 ± 1.09	-	0.59 ± 0.71	0.07 ± 0.16	0.40 ± 0.40	0.18 ± 0.19	-	-
**(*Z*)-β-ocimene ***	1.38 ± 2.03	1.08 ± 2.15	1.73 ± 0.85	1.56 ± 1.53	-	-	-	0.06 ± 0.15
**p-cymene**	-	-	2.69 ± 2.98	1.40 ± 0.63	-	-	-	-
**trans-linalool oxide (furanoid)***	-	-	-	0.04 ± 0.08	-	-	0.93 ± 0.91	0.15 ± 0.19
**limonene ***	1.76 ± 2.60	5.92 ± 4.32	4.77 ± 5.76	2.50 ± 3.51	-	-	1.95 ± 3.36	1.64 ± 2.12
**myrcene ***	-	-	0.90 ± 0.42	1.33 ± 0.72	-	-	0.14 ± 0.21	-
**sabinene**	2.36 ± 3.21	0.08 ± 0.15	2.71 ± 3.04	2.92 ± 2.17	0.28 ± 0.40	0.02 ± 0.03	1.10 ± 1.36	2.99 ± 5.21
**cyclolinalone**	-	-	-	-	-	-	-	-
**Sesquiterpenoids**								
**δ-guaiene**	-	-	-	-	-	-	-	-
**epi-prezizaene**	1.07 ± 1.91	-	-	-	-	-	-	-
**γ-elemene**	-	-	-	-	-	-	1.33 ± 0.73	1.21 ± 0.81
**δ-elemene**	2.58 ± 3.06	-	-	-	-	-	2.20 ± 0.96	1.81 ± 1.40
**germacrene A**	-	-	0.14 ± 0.22	0.28 ± 0.93	-	-	-	-
**germacrene D ***	-	-	2.66 ± 2.04	1.78 ± 2.42	0.03 ± 0.02	0.00 ± 0.00	-	-
**γ-terpinene**	-	-	7.84 ± 5.10	5.14 ± 3.61	-	-	-	-
**selina-4.11-diene**	-	-	-	-	-	-	-	-
**α-humulene***	-	-	1.71 ± 1.62	1.20 ± 1.71	0.32 ± 0.18	0.03 ± 0.03	0.19 ± 0.26	-
**α-ylangene**	-	-	0.29 ± 0.32	0.26 ± 0.40	0.03 ± 0.03	-	0.98 ± 2.19	0.16 ± 0.29
**β-caryophyllene ***	2.22 ± 1.99	-	2.15 ± 1.31	2.41 ± 2.35	1.35 ± 0.32	0.34 ± 0.35	0.98 ± 0.77	2.91 ± 2.47
**β-elemene ***	1.31 ± 1.58	-	1.14 ± 1.02	1.93 ± 4.94	0.00 ± 0.01	-	0.49 ± 0.68	4.38 ± 4.12
**bicyclogermacrene ***	-	-	0.54 ± 0.75	0.37 ± 0.62	-	-	-	-
**α-cis-bergamotene ***	-	-	0.42 ± 0.60	0.16 ± 0.27	-	-	0.20 ± 0.25	0.10 ± 0.27
**δ-cadinene**	-	-	1.55 ± 2.49	0.98 ± 1.74	0.04 ± 0.04	0.00 ± 0.00	0.51 ± 0.46	0.55 ± 0.50
**cyperene**	1.60 ± 3.15	-	-	-	-	-	-	-
**alloaromadendrene**	-	-	2.70 ± 2.13	1.58 ± 1.98	0.03 ± 0.03	-	1.01 ± 1.42	0.76 ± 0.83
**aromadendrene**	-	-	-	-	-	-	-	-
**β-bourbonene**	-	-	-	-	-	-	1.14 ± 1.18	-
**(*E-E*)-α-farnesene**	1.53 ± 1.97	-	0.21 ± 0.49	0.12 ± 0.24	-	-	±	-

**α-copaene ***	-	-	3.70 ± 3.08	2.19 ± 2.99	0.53 ± 0.50	0.04 ± 0.04	2.27 ± 2.69	4.98 ± 4.22
**α-cedrene**	0.92 ± 1.63	1.62 ± 2.51	±	0.12 ± 0.28	-	-	-	0.07 ± 0.19
**γ-muurolene**	-	-	0.08 ± 0.12	0.02 ± 0.07	0.01 ± 0.01	-	-	-
**α-selinene**	4.71 ± 8.95	-	0.17 ± 0.20	0.11 ± 0.17	0.05 ± 0.06	0.00 ± 0.01	-	-
**α-muurolene**	-	-	0.26 ± 0.52	0.10 ± 0.19	0.02 ± 0.02	-	-	-
**α-cubebene ***	-	-	0.08 ± 0.17	0.03 ± 0.07	-	-	-	-
**α-trans-bergamotene**	2.79 ± 2.93	-	3.15 ± 4.06	2.75 ± 3.49	-	-	-	-
**Carotenoid derivatives**								
**6-methyl-5-hepten-2-ol**	-	-	-	-	-	-	0.28 ± 0.43	1.10 ± 2.15
**6-methyl-3-hepten-2-one**	0.96 ± 1.27	3.28 ± 6.36	-	-	-	-	-	-
**Shikimic compounds**								
**benzaldehyde**	-	0.50 ± 1.01	-	-	-	-	1.11 ± 1.09	0.45 ± 0.43
**benzoic acid**	-	-	-	-	-	-	-	2.10 ± 4.17
**4-ethylanisole**	-	-	1.54 ± 2.59	1.85 ± 3.21	-	-	-	-
**4-methylanisole**	-	-	-	-	93.57 ± 1.75	98.36 ± 1.90	-	-
**indole**	-	-	0.23 ± 0.28	0.43 ± 0.96	1.58 ± 1.78	0.74 ± 1.59	1.40 ± 3.13	0.01 ± 0.04
**benzyl alcool**	-	-	-	-	-	-	-	1.42 ± 1.68
**eugenol**	-	-	-	-	-	-	5.71 ± 5.96	0.33 ± 0.42
**2-phenylethanol**	-	-	-	-	-	-	3.91 ± 2.28	0.46 ± 0.64
**guaiacol**	-	-	-	-	-	-	0.36 ± 0.53	1.65 ± 3.48
**Unknown**								
**NI (in *F. exasperata*)**	-	-	1.73 ± 3.95	0.17 ± 0.32	-	-	-	-
**NI (in *F. auriculata*)**	1.98 ± 2.50	-	-	-	-	-	-	-
**NI (in *F. fistulosa*)**	-	-	-	-	-	-	-	-
**NI (in *F. fistulosa*)**	-	-	-	-	-	-	-	-
**NI (in *F. fistulosa*)**	-	-	-	-	-	-	-	-
**NI (in *F. fulva*)**	-	-	-	-	-	-	-	-

**NI (in *F. septica*)**	-	-	-	-	-	-	1.87 ± 4.01	-

Cont.

**Compounds**	**Synchronous species**					
	Ficus fistulosa		***Ficus fulva***		***Ficus hispida***	
	**Female**	**Male**	**Female**	**Male**	**Female**	**Male**
**Fatty acid derivatives**						
**2-heptanone**	-	-	-	-	1.46 ± 3.85	-
**2-hexyl acetate**	-	-	-	-	-	-
**tridecanol**	-	-	-	-	-	-
**undecanal**	-	-	-	-	-	-
**undecane ***	-	-	-	-	-	0.01 ± 0.03
**nonanal ***	-	-	-	-	-	-
**decanal ***	0.98 ± 1.76	0.35 ± 0.34	-	-	-	-
**tridecane ***	-	-	-	-	0.08 ± 0.16	0.07 ± 0.16
**tetradecane ***	-	-	-	-	0.20 ± 0.32	0.45 ± 0.55
**(Z)-3-hexenol**	0.88 ± 1.96	2.27 ± 2.20	0.11 ± 0.10	0.68 ± 0.88	-	-
**(E)-2-hexenyl acetate**	-	-	-	-	-	-
**(Z)-3-hexenyl acetate ***	-	-	-	-	0.19 ± 0.36	0.39 ± 0.96
**Monoterpenoids**						
**cis-linalool oxide (furanoid) ***	4.32 ± 8.10	0.99 ± 0.62	1.05 ± 1.68	0.12 ± 0.14	1.92 ± 1.47	0.88 ± 1.07
**linalool ***	1.18 ± 1.42	4.17 ± 3.77	0.07 ± 0.05	0.30 ± 0.22	4.78 ± 6.02	3.47 ± 4.74
**(*E*)-β-ocimene ***	6.06 ± 5.02	12.25 ± 12.96	4.12 ± 4.71	1.80 ± 2.11	32.55 ± 19.07	28.06 ± 23.31
**α-pinene ***	0.57 ± 1.28	1.63 ± 3.61	2.53 ± 1.64	4.74 ± 4.93	3.16 ± 2.34	4.52 ± 5.07
**β-pinene ***	-	-	0.89 ± 0.60	2.74 ± 2.67	1.92 ± 2.23	1.17 ± 1.12
**1.8-cineole ***	4.15 ± 2.58	3.83 ± 2.82	0.14 ± 0.10	0.29 ± 0.15	0.06 ± 0.16	0.49 ± 1.05

perillene	3.56 ± 3.19	8.63 ± 11.39	0.57 ± 0.41	0.17 ± 0.15	0.94 ± 0.67	0.29 ± 0.38
(*Z*)-β-ocimene *	1.48 ± 3.30	0.03 ± 0.08	1.28 ± 0.36	1.22 ± 0.90	1.97 ± 1.27	1.20 ± 1.20
p-cymene	-	-	-	-	0.18 ± 0.26	1.12 ± 1.83
trans-linalool oxide (furanoid)*	1.51 ± 2.29	0.89 ± 1.15	0.26 ± 0.51	0.12 ± 0.13	0.28 ± 0.36	0.23 ± 0.35
limonene *	0.89 ± 1.17	1.60 ± 1.42	0.41 ± 0.29	0.35 ± 0.35	3.83 ± 7.36	4.27 ± 8.57
myrcene *	0.73 ± 1.19	0.37 ± 0.45	0.63 ± 0.19	0.69 ± 0.71	0.48 ± 0.49	0.44 ± 0.51
sabinene	1.13 ± 0.80	2.23 ± 1.46	1.05 ± 1.18	0.48 ± 0.19	0.41 ± 0.91	0.46 ± 0.72
cyclolinalone	-	-	-	-	2.13 ± 1.93	2.34 ± 3.05
**Sesquiterpenoids**						
δ-guaiene	0.51 ± 0.67	0.32 ± 0.65	0.99 ± 0.53	1.71 ± 0.56	-	-
epi-prezizaene	-	-	-	-	-	-
γ-elemene	-	-	-	-	-	-
δ-elemene	3.04 ± 2.89	2.85 ± 2.31	0.26 ± 0.05	0.15 ± 0.08	0.06 ± 0.24	±
germacrene A	-	0.26 ± 0.40	0.95 ± 0.13	0.91 ± 0.20	0.85 ± 0.92	0.32 ± 0.44
germacrene D *	10.44 ± 8.76	14.32 ± 12.14	3.25 ± 0.69	3.01 ± 0.70	1.67 ± 1.42	2.65 ± 4.25
γ-terpinene	-	-	-	-	0.30 ± 0.51	0.95 ± 2.07
selina-4.11-diene	-	-	-	-	1.97 ± 1.77	1.70 ± 1.71
α-humulene*	1.68 ± 1.73	1.27 ± 1.10	7.44 ± 1.13	7.26 ± 1.09	2.23 ± 1.62	2.26 ± 1.59
α-ylangene	-	-	-	-	0.37 ± 0.36	2.66 ± 4.21
β-caryophyllene *	6.46 ± 5.58	6.82 ± 5.16	53.16 ± 4.44	52.84 ± 8.14	7.31 ± 4.20	6.52 ± 3.67
β-elemene *	2.31 ± 2.21	3.53 ± 4.77	5.70 ± 0.62	5.16 ± 1.00	1.60 ± 1.54	0.50 ± 0.47
bicyclogermacrene *	2.59 ± 1.79	1.54 ± 1.58	1.36 ± 0.44	1.06 ± 0.87	1.86 ± 1.21	0.76 ± 0.79
α-cis-bergamotene *	1.61 ± 1.20	1.00 ± 0.49	-	-	0.23 ± 0.51	0.49 ± 1.22
δ-cadinene	-	-	1.23 ± 0.13	1.35 ± 0.45	2.02 ± 1.23	2.65 ± 1.58
cyperene	-	-	-	-	-	-
alloaromadendrene	2.59 ± 2.75	2.77 ± 5.45	0.35 ± 0.20	0.38 ± 0.26	0.30 ± 0.29	0.34 ± 0.43
aromadendrene	5.28 ± 3.56	4.64 ± 4.62	0.05 ± 0.01	0.03 ± 0.03	0.22 ± 0.38	0.37 ± 0.53
β-bourbonene	1.93 ± 2.10	1.32 ± 1.61	0.26 ± 0.05	0.27 ± 0.21	-	-
(*E-E*)-α-farnesene	4.83 ± 5.01	1.43 ± 1.48	-	-	0.27 ± 0.42	0.25 ± 0.39
α-copaene *	20.38 ± 14.50	9.91 ± 7.40	6.18 ± 1.01	6.37 ± 1.99	6.59 ± 4.35	9.39 ± 6.25

α-cedrene	-	-	-	-	-	-
γ-muurolene	-	-	-	-	2.17 ± 1.59	2.13 ± 2.13
α-selinene	0.62 ± 0.44	1.66 ± 2.02	0.41 ± 0.22	0.41 ± 0.05	0.43 ± 0.52	0.34 ± 0.32
α-muurolene	-	-	0.15 ± 0.06	0.19 ± 0.03	0.67 ± 0.41	1.10 ± 1.78
α-cubebene *	0.55 ± 0.49	0.54 ± 0.55	0.92 ± 0.09	0.87 ± 0.18	-	-
α-trans-bergamotene	-	-	-	-	4.20 ± 5.72	4.79 ± 4.76
**Carotenoid derivatives**						
6-methyl-5-hepten-2-ol	-	-	-	-	-	-
6-methyl-3-hepten-2-one	-	-	-	-	-	-
**Shikimic compounds**						
benzaldehyde	0.18 ± 0.25	0.22 ± 0.34	0.17 ± 0.34	0.06 ± 0.04	-	-
benzoic acid	-	-	-	-	-	-
4-ethylanisole	-	-	-	-	-	-
4-methylanisole	-	-	-	-	-	-
indole	-	-	-	-	1.69 ± 1.20	4.40 ± 6.69
benzyl alcool	-	-	-	-	-	0.18 ± 0.36
eugenol	-	-	-	-	-	-
2-phenylethanol	-	-	-	-	-	-
guaiacol	-	-	-	-	-	-
**Unknown**						
NI (in *F. exasperata*)	-	-	-	-	-	-
NI (in *F. auriculata*)	-	-	-	-	-	-
NI (in *F. fistulosa*)	1.40 ± 1.98	0.43 ± 0.66	-	-	-	-
NI (in *F. fistulosa*)	1.02 ± 2.09	0.12 ± 0.25	-	-	-	-
NI (in *F. fistulosa*)	1.53 ± 1.38	1.36 ± 1.17	-	-	-	-
NI (in *F. fulva*)	-	-	1.11 ± 1.65	0.20 ± 0.21	-	-
NI (in *F. septica*)	-	-	-	-	-	-
